# Vascular normalization confers an endothelial immune suppressive gene expression profile

**DOI:** 10.1002/1878-0261.70355

**Published:** 2026-10-30

**Authors:** Liqun He, Michael Welsh

**Affiliations:** ^1^ Department of Immunology, Genetics and Pathology Uppsala University Sweden; ^2^ Medical Cell Biology Uppsala University Sweden

**Keywords:** angiogenesis inhibition, breast cancer, immune checkpoint therapy, immune suppression, vascular endothelial growth factor, vascular normalization

## Abstract

The efficacy of immunotherapy is commonly increased by simultaneous anti‐angiogenesis treatment. An experimental breast cancer model exhibits vascular normalization with reduced vascular leakage when grown in mice with endothelial *Shb* deficiency. This mutant ablates the effects of vascular endothelial growth factor A on angiogenesis and leakage. Unexpectedly, the endothelial gene expression changes that were observed under these conditions suggested an immunosuppressive state. Comparison of healthy human breast endothelium with breast cancer endothelium revealed similar immune suppressive gene expression differences, and lung cancer and colorectal cancer showed analogous patterns of changes. These findings suggest that normalized or physiologically normal endothelium may have immunosuppressive features, with implications for the optimization of cancer immunotherapy regimens.

## Graphical abstract

We compare the immunoregulatory gene expression profiles of healthy endothelial cells or endothelial cells from tumors having a normalized endothelium (low leakage) with that of wild‐type tumors (high leakage) and observe that the wild‐type tumor endothelium exhibits immunostimulatory properties. This observation may have relevance for improving immunotherapy in combination with angiogenesis inhibition.

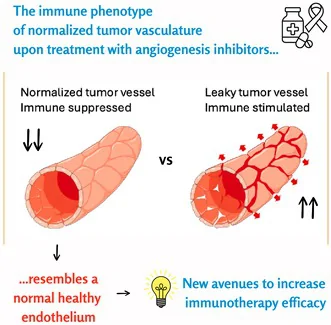

**Abbreviations**
SHB: *Src* homology 2 domain protein B
Treg: T regulatory cells
VEGFA: vascular endothelial growth factor A
VEGFR2: vascular endothelial growth factor receptor 2

Vascular normalization is a common response to treatment with angiogenesis inhibition in cancer therapy, leading to increased tumor vascular perfusion and reduced vessel leakage [1, 2]. The predominant view is that vascular normalization facilitates antitumor immunity because angiogenesis inhibitors often enhance the efficacy of immunotherapy when both are administered in combination [3, 4, 5, 6, 7]. However, recent studies on breast cancer endothelial function suggest that healthy or normalized endothelium may be predominantly immune suppressive [8, 9, 10]. Inhibition of vascular endothelial growth factor A (VEGFA) signaling is commonly used to achieve angiogenesis inhibition and vascular normalization [1]. The adapter protein *Src* homology 2 domain protein B (SHB), which is transcribed from the *Shb‐*gene operates downstream of vascular endothelial growth factor receptor 2 (VEGFR2) and is necessary for VEGFA‐induced angiogenesis and leakage [11]. Its primary mode of action appears to relay VEGFR2 signals to focal adhesion kinase [12], thus modifying endothelial cell properties. Consequently, the endothelial conditional *Shb* knockout was employed as an experimental model to study breast cancer metastasis and vascular and immune cell functions [10]. As anticipated, this experimental model exhibited vascular normalization but unexpectedly, this was accompanied by an immune suppressive phenotype associated with increased metastasis and increased infiltration of T regulatory cells (Tregs) [8, 10]. When the data were explored more in detail, the tumor immune suppressive immune cell profile was consistent with a reprogrammed endothelial gene expression profile as a consequence of endothelial *Shb* deficiency [9]. This conclusion was based on endothelial gene expression changes of genes belonging to the gene ontology categories ‘positive regulation of T cell activation’, ‘activation of immune response’, and ‘cytokine activity’. The analysis was performed by 10 different gene expression comparisons: (1) E0771 breast cancer endothelium relative to *Shb* endothelial knockout (normalized) breast cancer endothelium, (2) breast cancer arterial endothelium relative to healthy breast arterial endothelium, (3) breast cancer capillary endothelium relative to healthy breast capillary endothelium, (4) breast cancer venous endothelium relative to healthy breast venous endothelium, (5) lung cancer (non‐small cell) arterial endothelium relative to healthy lung arterial endothelium, (6) and (7) lung cancer type I and II capillary endothelium relative to healthy lung capillary endothelium, (8) lung cancer venous endothelium relative to healthy lung venous endothelium, (9) colorectal cancer endothelium relative to healthy colorectal endothelium, and (10) abnormal endothelial cells from human atherosclerotic plaques relative to normal endothelial cells. The immune suppressing endothelial gene expression alterations included reduced expression of genes coding for inflammatory cytokines or chemokines and altered expression of cell surface‐expressed immune regulating proteins [9]. The immune suppressive consequences of the altered endothelial gene expression profile correlated with reduced activity of inflammatory macrophages, more anti‐inflammatory CD163^+^ macrophages, fewer antigen‐presenting dendritic cells, and more Tregs [8]. The question remained as to whether these changes were representative for vascular normalization in general or specific for this experimental model. To address this, we compared the endothelial gene expression changes for immunomodulatory genes in tumors grown in *Shb‐*deficient endothelium relative to those grown in wild‐type mice with those of healthy human breast endothelial cells relative to breast cancer endothelial cells [9]. We reasoned that if vascular normalization restores endothelial features toward a nonpathological state, normalized endothelium should share transcriptional profiles with healthy endothelium. This was indeed found to be the case in line with the suggestion that they share immunomodulatory patterns. The changes in immune suppressive endothelial gene expression of healthy endothelium relative to disease‐associated endothelium were not unique to breast cancer but could also be observed in lung and colorectal cancer [9]. The most apparent changes were noted with respect to expression of inflammatory cytokines or chemokines that were expressed at lower levels in both healthy and normalized endothelium (Table 1). Particularly, healthy relative to tumor arterial endothelial cells exhibited a close similarity with *Shb* knockout normalized relative to wild‐type tumor endothelial cells, and this comparison was extended by comparison with atherosclerotic plaque endothelium. Atherosclerotic plaques contain proangiogenic and endothelial–mesenchymal transition (endMT) populations that normally do not exist in arteries and these exhibited an immunostimulatory profile compared with standard endothelial cells. A recent study has reported immune stimulatory functions of atherosclerotic plaque endothelial cells thus strengthening the overall concept of disease endothelium commonly being immune stimulatory [13].

**Table 1:** Summary of gene expression changes reported in [8, 9] of relevance to the immune promoting phenotype *Shb* iECKO normalized breast cancer endothelial cells. Also listed are gene expression changes showing a high degree of agreement between normalized breast endothelium in the experimental model of mouse breast cancer and healthy human endothelium relative to tumor endothelium.

Secreted proteins	Cell surface proteins	Immune regulating proteins
**Immune suppressive changes exerted by *Shb* iECKO normalization**		
*Bmp2*	*Axl*	*Cd36*
*Bmp4*	*Ccr4*	*Cd44*
*Bmp7*	*Dll4*	*Cd63*
*C2*	*Efnb1*	*Cd82*
*C1qbp*	*Jag1*	*Cd83*
*Cfh*	*Ltbr*	*Cd86*
*Cxcl12*	*Pecam1*	*Cd5l*
*Hgf*	*Sema3a*	*Icosl*
*Il24*	*Sema4b*	*Lgals9*
*Msmp*		*Sell*
*Pdgfd*		
*Tnfsf4*		
*Vegfa*		
*Vwf*		
**Concordance between healthy endothelium and normalized endothelium**		
*BMP4*	*AXL*	*CD36*
*C1QBP*	*DLL4*	*CD44*
*CFH*	*EFNB1*	*CD63*
*HGF*	*LTBR*	*CD82*
*IL24*	*SEMA4B*	*JAM2*
*MSMP*		*LGALS9*
*PDGFD*		
*VEGFA*		

The observed immune suppressive state of the normalized endothelium did not appear to involve expression of genes coding for proteins serving as conduits for leukocyte transendothelial migration but rather genes coding for cytokines or chemokines and immune suppressive transmembrane proteins that will skew the tumor immune cell microenvironment to an immune suppressive state [9]. However, examples of tumor endothelium that reduces leukocyte passage, thus creating an immune restricted environment, have been observed [14, 15], in line with the diversity of tumor phenotypes. It has been postulated that the immune suppressive feature of vascular normalization follows a biphasic pattern with a partly normalized endothelium that promotes immune responses and a fully normalized endothelium that restricts immune cell infiltration [14], and this could in part explain our current findings.

At present, the relevance of the tumor's endothelial cell properties for its immune status remains unresolved. On the one hand, the tumor endothelium shows ‘inflammatory’ properties. On the other hand, the tumor endothelium is assumed to be immune suppressive [3]. Our present report suggests that in many cases, even at stages when the tumor has developed, the endothelium exhibits immunostimulatory features and this may have consequences for therapy. A summary of the data supporting the hypothesis of this ‘viewpoint’ is presented in Fig. 1, which illustrates the dichotomy in gene expression profiles between the normalized and wild‐type tumor endothelium in extreme cases. Healthy endothelium appears to resemble normalized endothelium in many respects in their immunomodulatory gene expression pattern. Also notable is that the similarity between normalized and healthy endothelium is stronger for breast cancer than for lung cancer [9]. The overall pattern of immunostimulatory functions of disease endothelium agrees with a physiological need for immune activation in response to an insult, exemplified by the need to obtain an appropriate reaction to an infection. The initial endothelial response to early tumor development can be hypothesized to follow the same pattern. However, the issue of tumor immune suppression as a necessity for long term tumor expansion is contradicted by the apparent initial endothelial immune activating response. The results raise the possibility that other, nonendothelial tumor‐intrinsic immunomodulatory mechanisms that gradually develop during the evolution of the tumor underlie such immune suppressive responses. We suggest that in many cases, these are thus not a consequence of an inherent immune suppressive endothelial phenotype but rather tumor‐derived cues that shape an immune suppressive microenvironment consisting of tumor cells, fibroblasts, immune cells, and other cells independently of the endothelium. In addition, the commonly observed improved efficacy of combining immune therapy with angiogenesis inhibition is not incompatible with an immune suppressed state of normalized endothelium since an increased infiltration of Tregs [8] is likely to increase the efficacy of immunotherapy. Furthermore, other stroma components as suggested above evolve as a consequence of normalization and these may contribute to increased efficacy of immunotherapy. Better understanding of the mechanisms behind the immune suppressive mode of normalized endothelium may present further avenues for improving immunotherapy. Ideally, a regimen of angiogenesis inhibition that simultaneously is immune stimulatory by various means such as for example decreased endothelial expression of immune checkpoint inhibitors, increased endothelial expression of inflammatory cytokines, and expression of endothelial cell surface proteins that activate immune responses could be combined with immunotherapy to increase its efficacy. A potential candidate to study in this context is endothelial focal adhesion kinase since SHB plays a role in the regulation of its activity in response to VEGF.

**Fig. 1:**
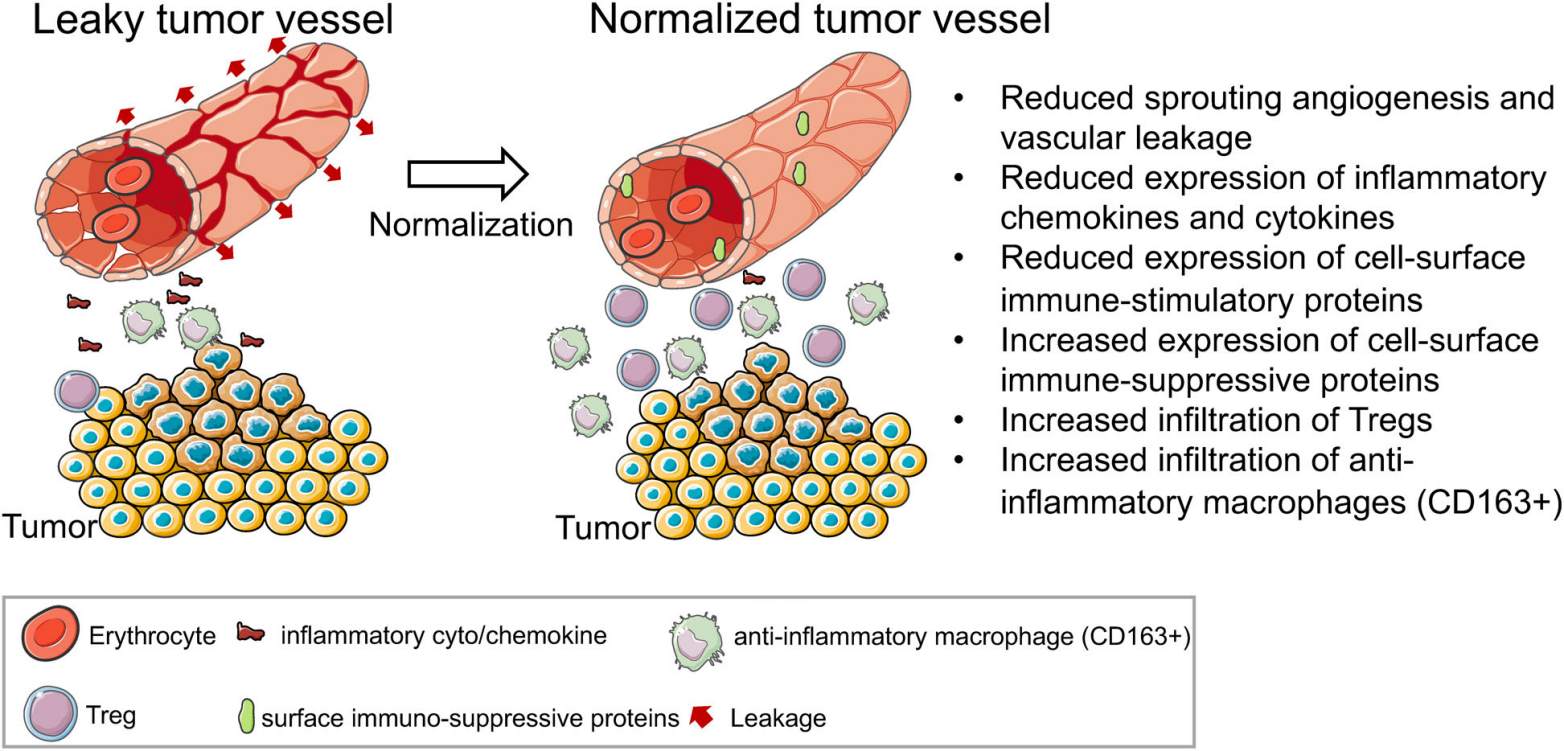
Model illustrating differences between normalized tumor endothelium due to endothelial *Shb* deficiency and wild‐type tumor endothelium. The normalized endothelium shows many similarities with healthy endothelium. A nonleaky (normalized tumor) versus a leaky vessel (wild‐type tumor) are shown. Presence of leakage, anti‐inflammatory (CD163^+^) macrophages, T regulatory cells (Tregs), inflammatory cytokine, or chemokine expression produced in endothelium and expression of immune suppressive cell surface proteins are shown as indicated in the legend. Illustrations were adapted from Servier Medical Art (https://smart.servier.com) licensed under CC BY 4.0 (https://creativecommons.org/licenses/by/4.0/).

The following limitations are acknowledged: The study provides correlative and not causal relationships that need to be functionally validated; the number of significant gene changes of relevance to the immune system was limited, and some of the genes exert context dependent effects; a limited number of tumors and datasets were investigated; the study does not take into account tumor heterogeneity, and we are not able to provide a clear strategy for achieving angiogenesis inhibition that is immune stimulatory.

## Conclusions

Both normalized endothelium and healthy endothelium exhibit gene expression changes that support an immune suppressed state compared with wild‐type breast cancer, lung cancer, and colorectal cancer endothelium. This notion may have consequences for improving anti‐angiogenic treatment with immunotherapy.

## Conflict of interest

The authors declare no conflicts of interest.

## Author contributions

MW and LH designed the study. LH performed bioinformatic analysis. MW and LH analyzed data. MW wrote the first version of the manuscript. LH provided comments and suggestions for text revision.
